# Natural Resources: Australia’s War on Drought

**Published:** 2007-07

**Authors:** Graeme Stemp-Morlock

Watering the lawn and washing the car are quintessential parts of summer. Yet, in some parts of Australia these activities are illegal. That’s because water is fast becoming the most precious commodity Down Under, so precious that every major urban center is under heavy water restrictions, and scientists are scouring the land for more water.

The shortage results partly from changing weather patterns—rain is falling at different times of the year and in shorter bursts. Australia is uniquely vulnerable to climate change, given the proportion of its relatively small land mass to the ocean that surrounds it; sea surface variations such as those seen with the El Niño–Southern Oscillation have a more profound effect here. According to the governmental *State of the Environment 2006* report, Australia—though a land of many climates—is overall the driest inhabited continent in terms of rainfall and streamflow.

Increased urbanization also plays a role. “The southeastern part of Australia where most people live is getting hotter and drier,” says Tom Hatton, director of the Water for a Healthy Country Flagship, the water research arm of Australia’s Commonwealth Scientific and Industrial Research Organisation (CSIRO). “If you project the demand of the five or six largest cities in the next twenty-five years, those cities are going to need up to forty percent more water, which we simply don’t have.”

CSIRO, in partnership with universities, governments, and private research agencies, has undertaken a massive project to find that water, spending AUS$80 million per year to find ways to increase the value that the country derives from water. One such innovation is the use of so-called Fleck sensors to monitor groundwater salinity, flow, and levels in virtual real time in a key agricultural area of Queensland. These wireless devices, which fit in the palm of a hand, feature long-range communication and solar recharging capabilities. The data they yield can be used, for example, to alert nearby sugarcane farmers if they are pumping too much ground-water before coastal seawater intrudes into the watershed.

The Fleck sensors are just one part of CSIRO’s larger Water Resources Observation Network (WRON). The goal of the network is to collect current data quickly and accurately. “To produce a national report on water resources using manual methods would be hugely costly, [and] takes about six months and an army of people producing reports that are almost instantly out of date,” says WRON manager Ross Ackland. “We need information systems that can produce something much quicker.”

The network is organizing all water information using a common framework. Some 100 to 200 different governmental, utility, industry, and research organizations currently collect water resource data, most of which is in proprietary formats. However, the new Web 2.0 technology of data aggregators—a news RSS feed is one well-known example—can help gather Australia’s water information together. Visitors to the WRON website can download a Mac or Windows “widget” that collects data on Australia’s dam levels and assimilates them into a standard format.

Although better management of water resources may help save water, it won’t create more potable water—but desalination plants can. That’s why the Water for a Healthy Country Flagship is also researching better ways to desalinate and recycle water. In May 2007, CSIRO and nine of Australia’s universities combined their research efforts to form the Advanced Membrane Technologies for Water Treatment Research Cluster, with the goal of reducing the energy requirements of desalination by up to 50% and developing new materials to improve membrane performance.

Many desalination plants force water through semipermeable membranes to reduce salts and contaminants. Over time, these contaminants build up on the membrane, requiring cleaning or replacing and more energy to force water through the membrane’s shrinking area. CSIRO is therefore exploring the use of membranes that bristle with carbon nanotube “forests,” which trap contaminants while letting water flow through almost without friction, allowing a lower-energy method of desalination.

The CSIRO Water Reuse Technologies project is also tackling the hot issue of water reuse and recycling with a little help from Mother Nature, injecting wastewater into underground aquifers to remove a variety of pathogens, endocrine disruptors, and disinfection by-products. The rate of contaminant decay depends on the geochemical composition and biotic communities present in the aquifer. One town south of Perth reuses sewage water for irrigation after it spends only 30 to 40 days in an aquifer. The lead scientist on the project, Simon Toze, says, “The phosphates are probably removed by chemical properties or absorbed by limestone, whereas most of the nitrogen and pathogens are taken out by organisms using the contaminants as a food source.” This practice not only improves water quality but may also give Australians a cheap and practical way to store water—a large concern as regular rainfall becomes more sporadic.

## Figures and Tables

**Figure:**
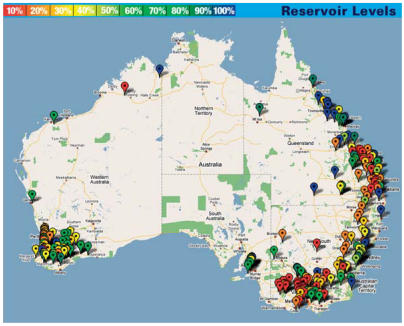
Mapping quest A national water data collection network can yield comprehensive data such as this “widget” readout of reservoir levels across Australia.

